# Screening methods for enzyme-mediated alcohol oxidation

**DOI:** 10.1038/s41598-022-07008-7

**Published:** 2022-02-22

**Authors:** Martina L. Contente, Irene Marzuoli, Hans Iding, Dennis Wetzl, Kurt Puentener, Steven P. Hanlon, Francesca Paradisi

**Affiliations:** 1 Department of Chemistry, Biochemistry and Pharmaceutical Sciences, University of Bern, Freistrasse 3, 3012 Bern, Switzerland; 2 Department of Food, Environmental and Nutritional Sciences (DeFENS), University of Milan, Via Celoria 2, 20133 Milan, Italy; 3 F. Hoffmann-La Roche Ltd, Process Chemistry and Catalysis (PCC), Grenzacherstrasse, 4070 Basel, Switzerland

**Keywords:** Biotechnology, Assay systems

## Abstract

Alcohol oxidation for the generation of carbonyl groups, is an essential reaction for the preparation of fine chemicals. Although a number of chemical procedures have been reported, biocatalysis is a promising alternative for more sustainable and selective processes. To speed up the discovery of novel (bio)catalysts for industrial applications, efficient screening approaches need to be established. Here, we report on an enzyme-mediated alcohol oxidation screening platform to rapidly detect the activities and selectivities of three classes of biocatalysts; ketoreductases (KREDs), alcohol oxidases (AlcOXs) and laccase-mediator systems (LMSs) with diverse substrates.

## Introduction

Alcohol oxidation to generate carbonyl moieties is one of the most common and important reactions in synthetic chemistry. Although a variety of chemical procedures were developed over the years, they mainly suffer from a difficult redox equivalent transport between reactants and oxidants. Traditionally, alcohol oxidation has been carried out with metal based catalysts^1–3^ or with metal-free approaches which often required chlorinated solvents^4,5^. Moreover, considering that the desired oxidation reaction only required the transfer of hydrogen (H^−^ and H^+^), the overall atom economy is dramatically poor. In the search for alternatives, biocatalysis plays a key role and various oxidation approaches using enzymes were developed^6–8^. Among the enzymes available for alcohol oxidations, the superfamily of ketoreductases (KREDs), alcohol oxidases (AlcOxs) and laccase-mediator systems (LMSs) are the most commonly employed. KREDs utilize oxidized nicotinamide cofactors (NAD(P)^+^) as hydride acceptor during the oxidation. The reversibility of the reaction explains the reason why KREDs can be used in both directions. In fact, the majority was employed for the enantioselective reduction of prochiral ketones^9,10^. The catalytic mechanism also implies that the oxidation of one equivalent of alcohol results in the consumption of one equivalent of NAD(P)^+^ forming its reduced form NAD(P)H. The high costs associated with these molecules led to the development of a variety of in situ regeneration methods (substrate-coupled or enzyme-coupled approaches) in order to use cofactors in catalytic amounts and only in their active form^11,12^. AlcOXs represent another class of enzymes useful for alcohol oxidation. Unlike reductases, they do not rely on nicotinamide cofactors but the reducing equivalents formed during the oxidation reaction, are transferred to molecular oxygen generating H_2_O_2_ in stoichiometric amounts^13,14^. Hydrogen peroxide is generally dismutated into O_2_ and H_2_O by the addition of a catalase. LMSs consist in organocatalytic systems typically using oxammonium species as oxidants. The *N*-oxide radical TEMPO, or its analogues, are oxidized by laccases into the corresponding reactive oxammonium species, while O_2_ is reduced to H_2_O. After the formation of the desired carbonyl compound, TEMPO is regenerated through the corresponding hydroxylamine form^15,16^. The main characteristics of these are described in Table 1.

**Table 1:** Summary of the characteristics of the 3 enzyme classes used in this study.

Enzyme class	Cofactor/mediator	Final hydride acceptor	Cofactor/mediator recycling approaches
Ketoreductase	NAD(P)^+^	NAD(P)^+^	NAD(P)H oxidase, acetone
Alcohol oxidase	None	O_2_	N/A
Laccase/mediator	TEMPO^a^	O_2_	Laccase

^a^For additional mediators used see Supplementary Information 1.

Improvement of biocatalysts requires the development of sensitive, efficient, and easy-to-implement high-throughput screening methods. Chromogenic and fluorogenic assays offer rapid results and are attractive methods for screening enzymes in high-throughput format^17^. Here we report on an enzyme-mediated alcohol oxidation screening platform (colorimetric and HPLC assays) to detect the activities of three classes of biocatalysts (KREDs, AlcOXs and LMSs).

## Results and discussion

**Figure 1:**
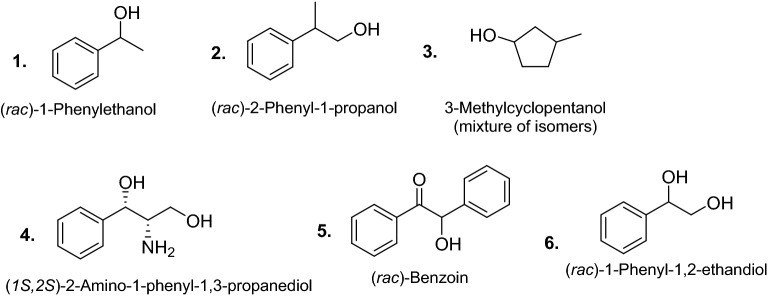
Substrates used in this study.

In this study, 6 model substrates have been selected (Fig. 1), including primary and secondary alcohols as well as diols. In addition, isopropanol, pure *(R)-***1**, and pure (*S)*-**1** were used in selected experiments. All screenings were performed in 96-plates (plate layouts and the corresponding screening results for every substrate/enzyme class are reported in the Supplementary Information 1, Sections 1, 5 and 7).

### Ketoreductase screening

**Figure 2:**
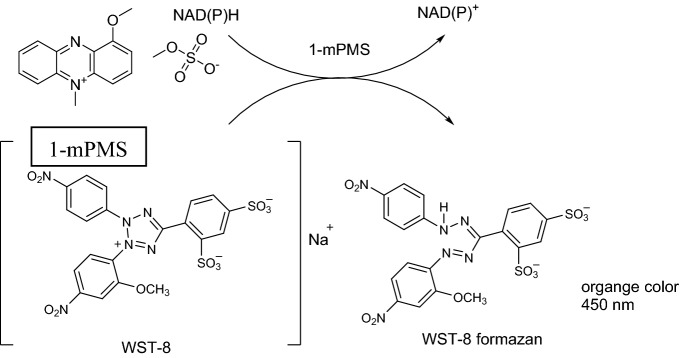
Schematic representation of the reaction for the detection of KRED activity using colorimetric WST-8 assay.

NAD(P)H absorbance at 340 nm is commonly used to measure the activity of KREDs^18,19^. Unfortunately, this approach is generally not suitable for high-throughput methods due to background noise deriving from the cell lysate. Colorimetric assays solve most of these problems and are quite amenable to high-throughput screening. Tetrazolium salts (*e.g.* nitroblue tetrazolium) were widely used for the determination of NAD(P)H, however, their application is limited by the low solubility of the formazan product^20^. Using a commercially available kit typically employed for cell viability (CCK-8), a quick screening of the proprietary Roche KRED library was carried out (220 KREDs). All the samples were used as cell-free extracts (CFEs); no enzyme purification was carried out. CCK-8 kit allows a convenient assay based on WST-8 (2-(2-methoxy-4-nitrophenyl)-3-(4-nitrophenyl)-5-(2,4-disulfophenyl)-2H-tetrazolium, monosodium salt). WST-8 in the presence of an electron mediator such as 1-methoxy-5-methylphenazium methylsulfate (1-mPMS), is readily reduced by NAD(P)H to produce an orange water-soluble formazan product, which can be detected by monitoring the absorbance at 450 nm (Fig. 2).

Among the advantages, CCK-8 is commercially available as single solution to add directly to the samples, no pre-mixing of components is required. Moreover, the detection sensitivity is higher than any other tetrazolium salts^21^. Although the potential of this assay was already described by Chamchoy and coworkers for the quantification of the activity of two model NAD(P)^+^—dependent dehydrogenases^21^, here the screening was tested with a sizable library of 220 different enzymes. Moreover, whereas the human G6DP (glucose-6-phosphate dehydrogenase) and the SDR (short chain dehydrogenase) from *Burkholderia pseudomallei* presented by Chamchoy et al*.*^21^ were employed in the reduction direction and required an enzyme-coupled approach for NA(P)H recycling, here the test reagents could be directly employed as regeneration system for the NAD(P)^+^ cofactor involved in the oxidation reactions (Fig. 2), which could therefore be used in low concentration (1:10 cofactor:substrate).

### KRED library

**Figure 3:**
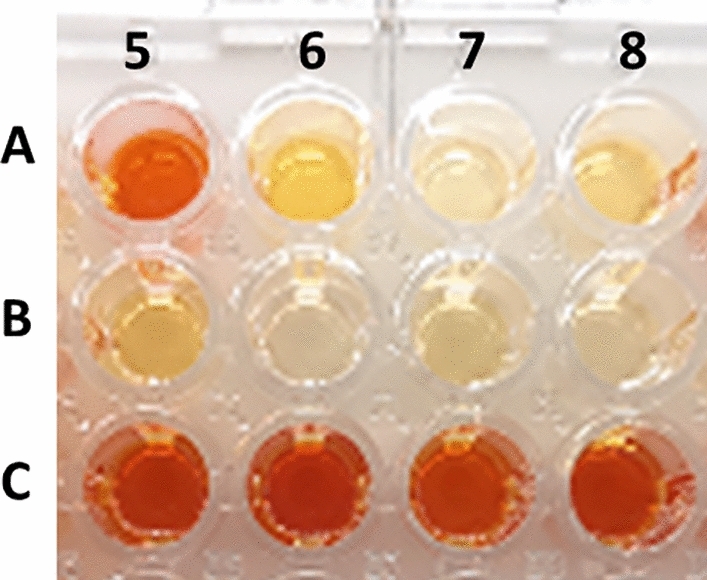
KRED assay at 24 h; A5: KP00104, A6: KP00127, A7: KP00128, A8: KP00129; B5: KP00138, B6: KP00139, B7: KP00140, B8: KP00141, C5: KP00150, C6: KP00151, C7: KP00152, C8: KP00153. Increased orange color intensity corresponds to higher absorbance values and consequently to a more active enzyme.

220 KREDs previously expressed in *E. coli* strains were prepared at Roche as CFEs and tested towards the 6 substrates in Fig. 1. The catalysts were divided in 3 groups: NAD^+^-, NADP^+^-dependent and Cofactor Undefined. The cofactor undefined plates were screened by adding separately NAD^+^ or NADP^+^ to elucidate their preferential cofactor dependency (See Supplementary Information 1, Section 1). Absorbance was monitored at different reaction times; 30 min, 2 h and 24 h, which allowed for the quick identification of the best hits in terms of activity and reaction rate for each substrate. As an example NADP^+^-dependent KREDs assayed with a racemic mixture of substrate **1** after 24 h incubation time are shown in Fig. 3, illustrating that the most active enzymes can clearly be distinguished. For full results with **1** and with the remaining substrates see Supplementary Information 1, Section 1. A handful of hits were found for all six substrates and the activity could be confirmed by HPLC analysis (See Supplementary Information 1, Section 2). The KRED library was also screened for the oxidation of isopropanol to form acetone. If this reaction is sufficiently reversible, acetone can be used as a sacrificial substrate for the regeneration of the cofactor in preparative-scale oxidation reactions.

### Evaluation of the stereoselectivity using (R)-1 or (S)-1

**Figure 4:**
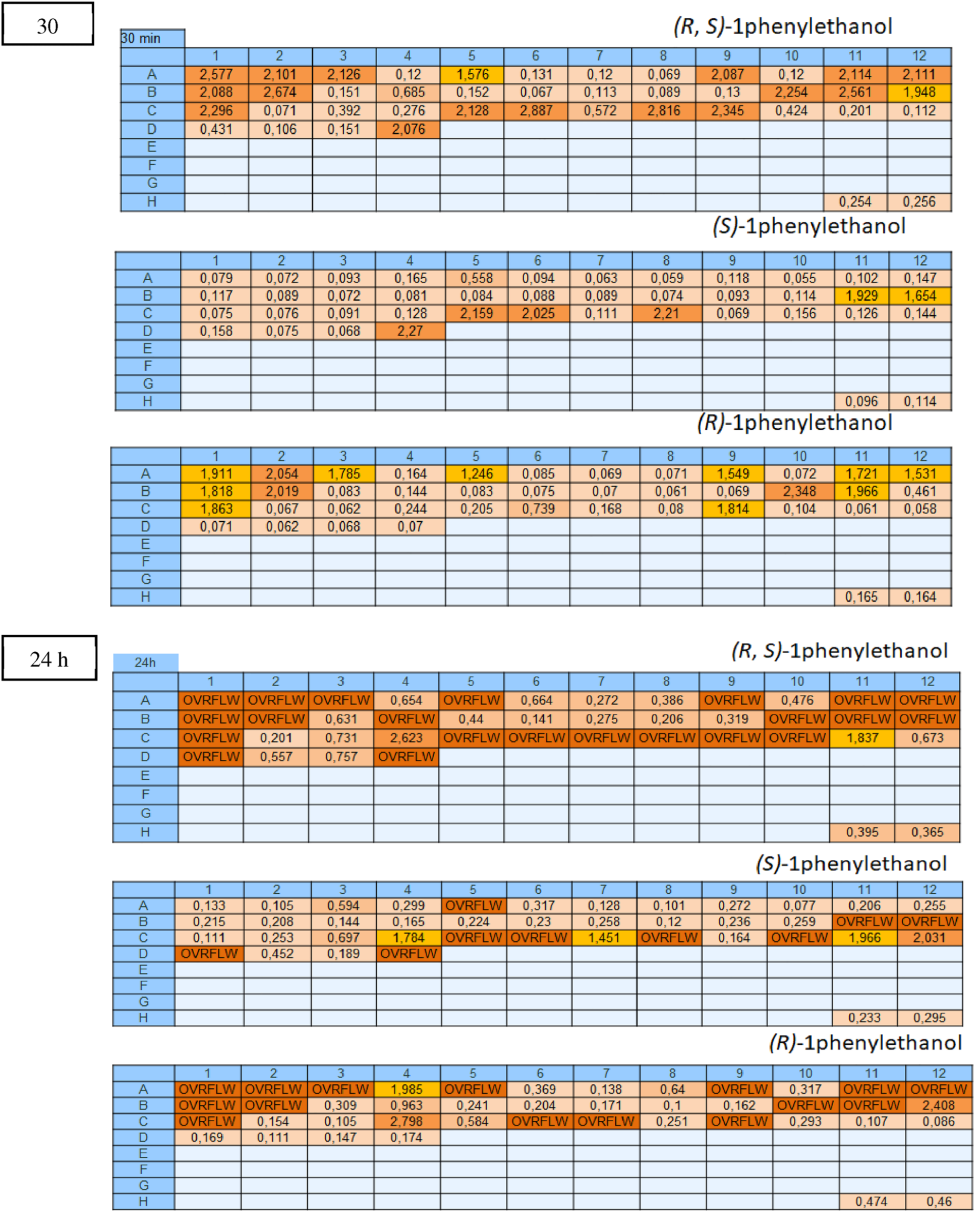
Comparison at 30 min and 24 h between results obtained by the addition of the racemic substrate **1** and the pure enantiomers (*(R)*-**1** and *(S)*-**1**). Increased orange color intensity corresponds to higher absorbance values (reported in the cells) and consequently to a more active enzyme. Ovrflw: absorbance values over 3 (corresponding to the limit of the instrument used for the detection).

For the evaluation of the stereoselectivity of the Roche KRED collection (NAD^+^, NADP^+^ and Cofactor Undefined enzymes), substrate **1** was used. A comparison between the results obtained by the addition of the racemic substrate **1** and the pure enantiomers (*(R)-***1** and *(S)-*) **1**) was subsequently carried out for each plate. As an example, the active NADP^+^-dependent KREDs (Fig. 4) with one of the enantiomers and both of them at 30 min and 24 h are reported in Table 2. While enzymes in position **C5**, **C8**, **C10**, **D1** and **D4** are selective for (*S*)-1-phenylethanol (results at 24 h), only **C5**, **C8** and **D4** demonstrated a higher reaction rate (increased orange color and absorbance at 30 min). The same comparison was performed for (*R*)-selective KREDs and enzymes active on both enantiomers. Interestingly, enzymes **A5** and **C6** showed a stereopreference for *(R)*-**1** and *(S)*-**1** respectively at the beginning of the oxidation reaction, but by 24 h both the enantiomers were converted. **B11** is the only enzyme showing high initial activity with both enantiomers. A full overview of KREDs’ screening is reported in the Supplementary Information 1, Section 3.

**Table 2:**
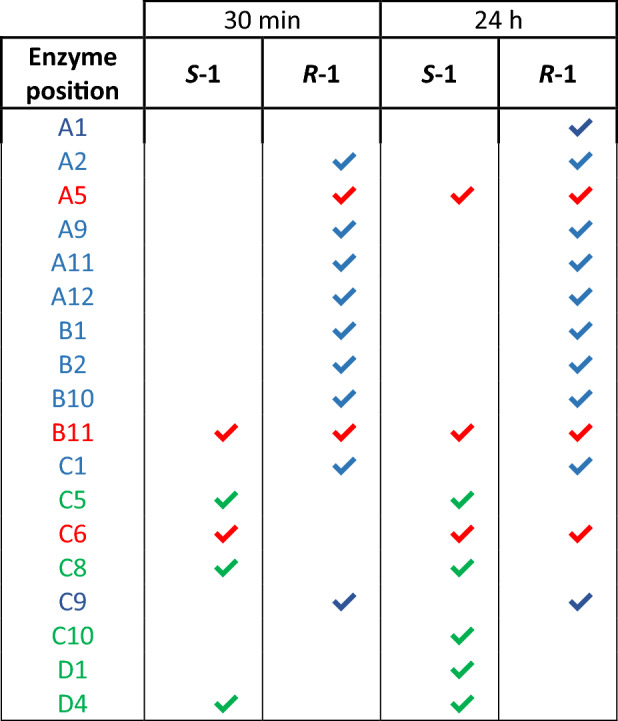
Stereopreference evaluation of NADP^+^-dependent KREDs.

Summary of NADP^+^-dependent enzymes active on *(R)*-**1** in blue, *(S)*-**1** in green and both the enantiomers in red.

### Alcohol oxidases

The activity of commercial AlcOXs, 10 from Gecco and 2 from Sigma Aldrich (Supplementary Information 1, Section 5) were assayed using a commercial kit (MAK 311) for the detection of H_2_O_2_, formed during alcohol oxidation. It provides a simple and adaptable assay for quantitative determination of H_2_O_2_ concentration without any sample pre-treatment. This test utilizes the chromogenic Fe^3+^—xylenol orange reaction, in which a purple complex is formed when Fe^2+^ is oxidized to Fe^3+^ by peroxides present in the sample, generating a colorimetric (585 nm) result, proportional to the level of peroxide present. While this assay was described for the evaluation of *para*-phenol oxidases, the catalysts were purified^22^. No data with complex CFEs were reported so far. Moreover, the optimized formulation reduces interference by substances in the crude enzyme samples.

The best conditions were found using GLUO (glucose oxidase from *Aspergillus niger*) with substrates **2** and **5** (1 mM) giving 32% and 7% molar conversion respectively (Table 3). These data were obtained by fitting the sample absorbance values (duplicate) to a calibration curve previously established following the kit instructions (Supplementary Information 1, Section 6). A reaction with higher substrate loading (10 mM), but unchanged enzyme concentration (1 mg/mL), and reaction time (2 h), was subsequently carried out. Based on the calibration curve, 16% and 9% molar conversion were observed for substrate **2** and **5** respectively.

**Table 3:** Activity of glucose oxidase (GLUO) with test substrates and glucose, employed as positive control.

Substrate	Abs. 585 nm
1	0.434
2	0.808
3	0.418
4	0.415
5	0.660
6	0.418
Negative control	0.410
Glucose	2.007
Glucose	2.007

Activity, as determined by Abs_585nm_ after 2 h, was observed with substrates 2 and 5 at 1 mM scale. As negative control the enzymatic reaction without the addition of any substrate has been utilized.

Due to lack of reactivity of the 10 Gecco enzymes towards the selected substrates, further investigations using the natural substrates eugenol and vanillyl alcohol of EUGO (Eugenol oxidase *Rhodococcus jostii*) and VAO (Vanillyl alcohol oxidase *Penicillium simplicissimum*) (Supplementary Information 1, Section 5) were carried out at 1 mM and 10 mM scale. In all cases full conversion was obtained between 2 and 24 h. It was suspected that possible catalase-mediated by-reactions (the Gecco enzymes are sold as *E. coli* CFEs) could interfere with the kit, rapidly converting the H_2_O_2_ produced by the oxidases and therefore making the detection highly unreliable. The reactions (all 6 substrates and 10 Gecco enzymes) at 1 mM scale were then monitored by HPLC (Supplementary Information 1, Section 4). 10% molar conversion was observed with substrate **2** and HMFO (5-Hydroxymethylfurfural oxidase *Methylovorus strain*) after 2 h (stable after 24 h). For substrate **5**, 22% molar conversion was obtained with NICO (6-Hydroxy-d-nicotine oxidase *Arthrobacter nicotinovorans*). None of the other substrates gave any detectable conversion.

### Laccase-mediator systems

For the laccase-mediator systems, a single enzyme laccase C from *Trametes sp.* was screened with different mediators (Supplementary Information 1, Section 7) and pH values (4.5–6.0). Different reaction conditions were found to be active for the oxidation of substrate **1** (90–93% molar conversion using AZADO as mediator at all pH values), **2** (60% molar conversion using TEMPO, pH 6) and **3** (65% molar conversion using AZADO, pH 4.5), no clear results have been obtained for substrate **4** and **6,** probably due over-oxidation phenomena, typically observed with the use of LMSs. No oxidation reaction was observed for substrate **5**.

## Conclusions

Among the screening methods employed in this study, the CCK-8 assay works efficiently with KRED enzymes, giving quick and reliable results for the alcohol oxidation of several substrates without the need for enzyme purification. No substrate- or enzyme- coupled method was necessary for the regeneration of cofactors which could be used in low concentration (1:10 cofactor:substrate). In contrast, MAK-311 was unsuitable for the detection of H_2_O_2_ in cell lysates likely due to background catalase activities originating from the expression host. Chromogenic or fluorogenic assays are the most attractive methods for monitoring catalysis in high-throughput formats; among them we selected only colorimetric assays for which reagents are commercially available and products easily detectable using commonly available instruments, or even visually (color change) in order to offer facile, practical solutions to laboratories working with enzymes as well as to new comers in the field.

For LMSs, HPLC/GC analysis remain the first choice, principally due to the multiple oxidation products which are often observed in laccase-mediated oxidation reactions. Taking into account results from all three enzyme classes the most amenable substrates for oxidation were substrates **1** and **3**, while **4** and** 6** were poorly converted in all cases. Regarding substrate **5**, low conversion was observed, possibly due to the low aqueous solubility of this substrate. No improvement was seen on addition of DMSO (1–10%) as cosolvent. The highest conversion (22%) was obtained using NICO AlcOx, from the Gecco kit at 1 mM scale. Substrate **2** was oxidized with maximum conversion using two enzymes belonging to the NAD-KRED library (92 and 90% molar conversion respectively after 24 h). The stereoselectivity was investigated only for the screening of the in-house KRED library with substrate **1.** The photometric/colorimetric assay described above allowed for a rapid evaluation of this property across both NAD- and NADP-dependent enzymes.

## Methods

### Materials

Commercially available reagents were purchased from Thermo Fischer Scientific or Merck (Sigma Aldrich). Organic solvents, chemical standards as well as CCK-8 kit and MAK 311 kit were bought from Merck (Sigma Aldrich). KRED-library was provided by Roche (F. Hoffmann-La Roche Ltd, Basel. Switzerland). Ten alcohol oxidases were purchased from Gecco and two from Merck (Sigma Aldrich) as reported in the Supplementary Information 1, Section 5. Laccase C from from *Trametes *sp*.* was purchased from ASA Spezialenzyme GmbH and the plate containing the different mediators (Supplementary Information 1, Section 7) provided by Roche (F. Hoffmann-La Roche Ltd, Basel. Switzerland). HPLC analyses have been performed using a C18 column (Xbridge, Waters), 254 nm, 0.8 mL/min gradient 95:5 water (+ 0.1%TFA)/ACN to 5:95 ACN/water (+ 0.1% TFA) in 10 min.

### KRED screening

Reaction conditions: 10 mM substrate, 1 mM cofactor (NAD^+^ or NADP^+^ depending on the plates), 10 µL crude extract (after centrifugation 3000 *g* × 10 min for Roche in-house KREDs, after resuspension in 1 mL of phosphate buffer 100 mM pH 7.0 for commercial enzymes), 25 °C. The Cofactor Undefined plate was screened adding separately NAD^+^ or NADP^+^ or, in the case of the evaluation of the stereoselectivity using (*R*)-**1** or (*S*)-**1**, both cofactors were added together. CCK-8 KRED-kit reagent (Sigma Aldrich) was added from the beginning of the reaction. Reaction volume 110 µL consisting of: 10 µL substrate (100 mM stock solution in phosphate buffer 100 mM pH 7.0 or DMSO depending on the solubility of the substrate), 10 µL cofactor (10 mM stock solution in phosphate buffer 100 mM pH 7.0), 10 µL crude extract as described above, 10 µL kit reagent, 70 µL phosphate buffer 100 mM pH 7.0. The plates were covered and incubated at 25 °C, no shaking was necessary. The increase in absorbance (corresponding to a more intense orange color) has been selected as criterion to determine the enzymatic performance in terms of activity and reaction rate. To check the reliability of the system some reactions were analyzed through HPLC using commercial products as reference (Supplementary Information 1, Section 2).

### AlcOx screening

Reaction conditions: 1 or 10 mM substrate, 1 mg/mL enzyme in phosphate buffer 50 mM pH 7.0, 28 °C, gently shaking for 2 h in 96 well plates. Reaction volume 100 µL composed of: 10 µL substrate (10 or 100 mM stock solution in phosphate buffer 50 mM pH 7.0), 10 µL enzyme (stock solution 10 mg/mL in phosphate buffer 50 mM pH 7.0), 80 µL phosphate buffer 50 mM pH 7.0. For substrate **5**, due to its poor aqueous solubility, a stock solution of 100 mM or 1 M in DMSO was used. The reactions were consequently composed of 1 µL substrate, 10 µL enzyme as previously described and 89 µL phosphate buffer 50 mM pH 7. After 2 h of reaction, the assay was prepared following the kit instructions and left to react for 30 min, after which the absorbance at 585 nm was measured. The product concentration was evaluated using a previously prepared calibration curve (Supplementary Information 1, Section 6).

### LMS biotransformations

To pre-filled plates containing 50 mM acetate buffer at different pH (4.5–6.0), 1 mM of different mediators (Supplementary Information 1, Section 7) and water were added the substrates at 10 mM concentration (100 µL of substrate, starting from a stock solution of 100 mM substrate dissolved in water or DMSO depending on the substrate solubility) and laccase C at 0.8 mg/mL (10 µL of enzyme, starting from a stock solution of 80 mg/mL in water). Final reaction volume 1 mL, 28 °C, gently shaking. The plates were closed with a gas-permeable membrane to let the oxygen pass through. The biotransformations were analyzed after 2 and 24 h through HPLC or GC following the analytical methodologies described in the Supplementary Information 1, Section 4.

## Supplementary Information

Supplementary Information.
