# Supplementary material for: Screening methods for enzyme-mediated alcohol oxidation
Source: Sci Rep. 2022 Feb 22;12:3019. doi: 10.1038/s41598-022-07008-7 (PMC8864024; doi:10.1038/s41598-022-07008-7)
Supplement: Supplementary file 1 — Supplementary Information. [file 41598_2022_7008_MOESM1_ESM.docx]

Supplementary Information

Screening methods for enzyme-mediated alcohol oxidation

Martina L. Contente^1,2,^ Irene Marzuoli^3^, Hans Iding^3^, Dennis Wetzl^3^, Kurt Puentener^3^, Steven P. Hanlon^3^* , Francesca Paradisi^1^*

^1^ Department of Chemistry, Biochemistry and Pharmacy, University of Bern, Freistrasse 3, 3012 Bern, Switzerland

^2^ Department of Food, Environmental and Nutritional Sciences (DeFENS), University of Milan, via Mangiagalli 25, 20133, Milan, Italy

^3^F. Hoffmann-La Roche Ltd, Process Chemistry & Catalysis (PCC), Bldg 62/136, 4070 Basel, Switzerland

**Contents**

1. In-house KRED screening results and plate layout p2
2. Evaluation of the KRED assay reliability p3
3. Evaluation of stereoselctivity using (*R*)-**1** or (*S*)-**1** for NAD^+^ and Cofactor Undefined p4

KREDs.

1. HPLC and GC analysis p8
2. Alcohol Oxidases p8
3. Calibration curve with increased concentration of H_2_O_2_ p9
4. LMS plate layout, table of mediators and results p9
5. **KRED screening results and plate layout**

| **NAD^++^** | 1 | 2 | 3 | 4 | 5 | 6 | 7 | 8 | 9 | 10 | 11 | 12 |
| --- | --- | --- | --- | --- | --- | --- | --- | --- | --- | --- | --- | --- |
| A | K00070 | K00071 | K00072 | K00073 | K00074 | K00075 | K00076 | K00077 | K00078 | K00079 | K00080 | K00081 |
| B | K00082 | K00083 | K00084 | K00085 | K00086 | K00087 | K00088 | K00089 | K00090 | K00091 | K00092 | K00093 |
| C | K00094 | K00095 | K00096 | K00097 | K00098 | K00099 | K00100 | K00101 | K00102 | K00103 | K00104 | K00105 |
| D | K00106 | K00107 | K00108 | K00109 | K00110 | K00111 | K00112 | K00113 | K00114 | K00115 | K00116 | K00117 |
| E |  |  |  |  |  |  |  |  |  |  |  |  |
| F |  |  |  |  |  |  |  |  |  |  |  |  |
| G |  |  |  |  |  |  |  |  |  |  |  |  |
| H |  |  |  |  |  |  |  |  |  |  | NK | NK |

BEST ENZYMES NAD^+^-KREDs: Substrate **1**: A1 (K00070); C3 (K00096); C9 (K00102)

Substrate **2**: C4 (K00097); D4 (K00109); D11 (K00116)

Substrate **3**: A10 (K00079); A12(K00081); B7 (K00088)

Substrate **4**: A2 (K00071); A5 (K00074); C4 (K00097)

Substrate **5**: B8(K00089); C11 (K00104); D7 (K00112)

Substrate **6**: B5 (K00110); C4 (K00097); D9 (K00114)

**Isopropanol**: A6 (K00075); B2 (K00083); B1 (K00082)

| **NADP^++^** | 1 | 2 | 3 | 4 | 5 | 6 | 7 | 8 | 9 | 10 | 11 | 12 |
| --- | --- | --- | --- | --- | --- | --- | --- | --- | --- | --- | --- | --- |
| A | KP00122 | KP00123 | KP00124 | KP00125 | KP00104 | KP00127 | KP00128 | KP00129 | KP00130 | KP00131 | KP00132 | KP00133 |
| B | KP00134 | KP00135 | KP00136 | KP00137 | KP00138 | KP00139 | KP00140 | KP00141 | KP00142 | KP00143 | KP00144 | KP00145 |
| C | KP00146 | KP00147 | KP00148 | KP00149 | KP00150 | KP00151 | KP00152 | KP00153 | KP00154 | KP00155 | KP00156 | KP00157 |
| D | KP00158 | KP00159 | KP00160 | KP00088 |  |  |  |  |  |  |  |  |
| E |  |  |  |  |  |  |  |  |  |  |  |  |
| F |  |  |  |  |  |  |  |  |  |  |  |  |
| G |  |  |  |  |  |  |  |  |  |  |  |  |
| H |  |  |  |  |  |  |  |  |  |  | NK | NK |

BEST ENZYMES NADP^+^-KREDs: Substrate **1**: A12 (KP00133); C8 (KP00153); C9 (KP00154)

Substrate **2**: B12 (KP0014); C8 (KP0015); C11 (KP00156)

Substrate **3**: A1(KP0012); A5 (KP00104); C4 (KP00149)

Substrate **4**: A5 (KP00104); C11 (KP00156)

Substrate **5**: A1 (KP0012); B5 (KP0013); B7(KP00140)

Substrate **6**: B12 (KP00145); C5 (KP00150); C9(KP00154)

**Isopropanol**: A9 (KP00130); C2 (KP00147); C4 (KP00149)

| **Cofactor Undefined** | 1 | 2 | 3 | 4 | 5 | 6 | 7 | 8 | 9 | 10 | 11 | 12 |
| --- | --- | --- | --- | --- | --- | --- | --- | --- | --- | --- | --- | --- |
| A | KX00001 | KX00002 | KP00161 | KX00004 | KX00005 | KX00006 | KX00007 | KX00008 | KX00009 | KX00010 | KX00011 | KX00012 |
| B | KX00013 | KX00014 | KX00015 | KX00016 | KX00017 | KX00018 | KX00019 | KX00020 | KX00021 | KX00022 | KX00023 | KX00024 |
| C | KX00025 | KX00026 | KX00027 | KX00028 | KX00029 | KX00030 | KX00031 | KX00032 | KP00162 | KP00163 | KX00035 | KX00036 |
| D | KP00164 | KX00038 | KX00039 | KX00040 | KX00041 | KX00042 | KX00043 | KX00044 | KX00045 | KX00046 | KX00047 | KX00048 |
| E | KX00049 | KX00050 | KX00051 | KX00052 | KX00053 | KX00054 | KX00055 | KX00056 | KX00057 | KX00058 | KX00059 | KX00060 |
| F | KX00061 | KX00062 | KX00063 | KX00064 | KX00065 | KX00066 | KX00067 | KX00068 | KX00069 | KP00165 | KX00071 | KX00072 |
| G | KX00073 | KP00166 | KX00075 | KX00076 | KX00077 | KX00078 | KX00079 | KX00080 | KX00081 | KX00082 | KX00083 | KX00084 |
| H | KX00085 | KX00086 | KP00167 | KX00088 | KX00089 | KX00090 | KX00091 | KX00092 |  |  | NK | NK |

BEST ENZYMES -Cofactor Undefined-KREDs with NAD^+^:

Substrate **1**: D10 (KX00046); G5 (KX00077); H1 (KX00085)

Substrate **2**: D11 (KX00047)

Substrate **3**: E6(KX00054); F12 (KX00072); H1 (KX00085)

Substrate **5**: C10 KP00163

Substrate **5**: -

Substrate **6**: B7 (KX00019); C11 (KX00047); E8 (KX00056)

**Isopropanol**: B8 (KX00020); B9 (KX00021)

BEST ENZYMES -Cofactor Undefined KREDs with NADP^+^-:

Substrate **1**: G2 (KP00166); G12 (KX00084); H8 (KX00092)

Substrate **2**: A7 (KX00007); C10 (KP00163); G2 (KP00166)

Substrate **3**: A6 (KX00006); F11 (KX00071); H8 (KX00092)

Substrate **4**: C10 (KP00163)

Substrate **5**: A3 (KP00161); B10 (KX00022)

Substrate **6**: C10 (KP00163); G2 (KP00166)

**Isopropanol**: A6 (KX00006); F12 (KX00072)

**2. Evaluation of the KRED assay reliability**

To check the reliability of the system, some of the biotransformations have also been analyzed by HPLC: C18 (Xbridge, Waters), 254 nm, 0.8 mL/min gradient 95:5 water (+0.1%TFA)/ACN to 5:95 ACN/water(+0.1% TFA) in 10 min.

**NAD^+^-KREDs**

- Sub. **1**, enzyme C3 (K00096). Rt substrate: 5.62 min, product: 5.99 min: 97% mc after 2 h
- Sub. **1**, enzyme C9 (K00102). Rt substrate: 5.62 min, product: 5.99 min: 98% mc after 2 h
- Sub. **2**, enzyme C4 (K00097). Rt substrate: 5.88 min, product: 5.99 min: 92% mc after 24 h
- Sub. **2**, enzyme D4 (K00109). Rt substrate: 5.88 min, product: 5.99 min: 90% mc after 24 h
- Sub. **2**, enzyme D11 (K00116). Rt substrate: 5.88 min, product: 5.99 min: 88% mc after 24 h
- Sub. **5**, enzyme C11 (K00116). Rt substrate: 6.18 min, product: 6.87 min: 10% mc after 24 h
- Sub. **5**, enzyme D7 (K00112). Rt substrate: 6.18 min, product: 6.87 min: 5% mc after 24 h

**NADP^+^-KREDs**

- Sub.. **1**, enzyme A12 (KP00133). Rt substrate: 5.62 min, product: 5.99 min: 95% mc after 24 h
- Sub. **1**, enzyme C8 (KP00153). Rt substrate: 5.62 min, product: 5.99 min: 97% mc after 24 h
- Sub. **1**, enzyme C9 (KP00154). Rt substrate: 5.62 min, product: 5.99 min: 96% mc after 24 h
- Sub. **2**, enzyme B12 (KP00145). Rt substrate: 5.88 min, product: 5.99 min: 38% mc after 24 h
- Sub. **2**, enzyme C8 (KP00153). Rt substrate: 5.88 min, product: 5.99 min: 15% mc after 24 h

Sub. **2**, enzyme C11 (KP00156). Rt substrate: 5.88 min, product: 5.99 min: 55% mc after 24 h

mc: molar conversion

**3. Evaluation of the stereoselectivity using (*R*)-1 or (*S*)-1 for NAD^+^- and Cofactor undefined-KREDs**

Comparison between the results obtained by the addition of the racemic mixture of **1** and the pure enantiomers (***R)*-1** and (***S)*-1** at 30 min and 24 h.

**NAD^+^-KREDs**

30 min


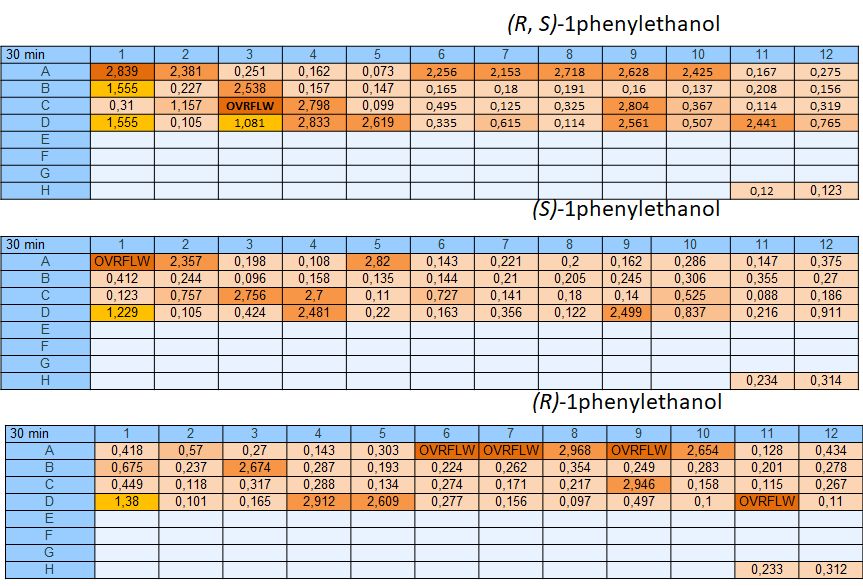

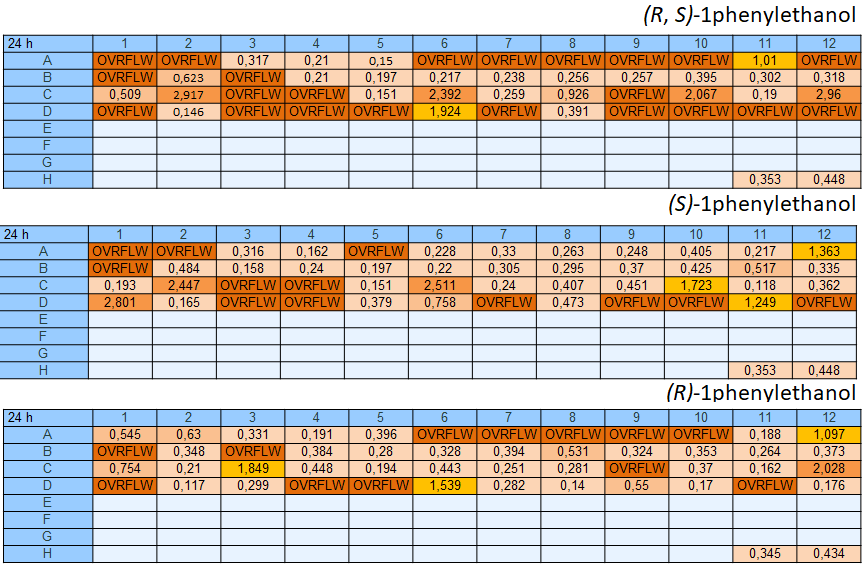


24 h

30 min


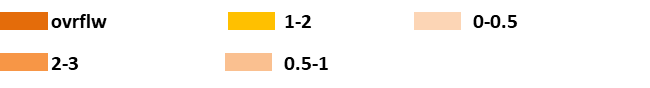


*ovrflw: absorbance values over 3 (corresponding to the limit of the instrument used for the detection).

After a comparison with the plate with the addition of racemic substrate **1** and 1 mM NAD^++^ at 30 min and 24 h we can conclude that:

**A1**, A2, C2, C4, C6, C10, D3, D7, D9, D10, D12 active towards ***S*-1**

**A6**, **A7**, A8, **A9**, A10, B3, C9, C12, D5 active towards ***R*-1**

C3 (stereopreference for ***S*-1**), A12, B1, D1, D4, D6, D11 active towards **both the enantiomers**

(In bold are reported the faster reactions)

**Cofactor Undefined-KREDs (NAD^+^)**


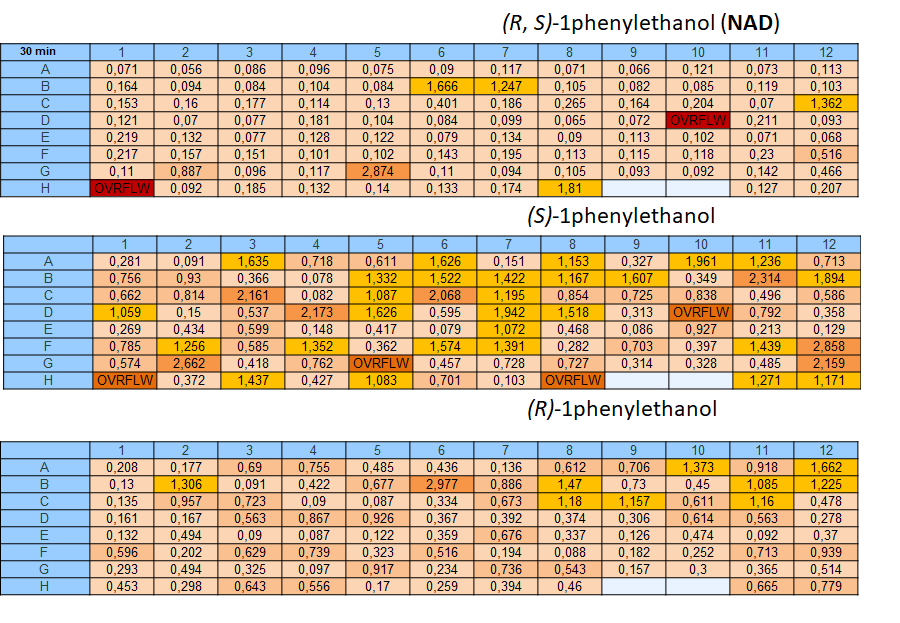


30 min


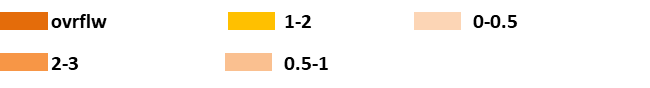


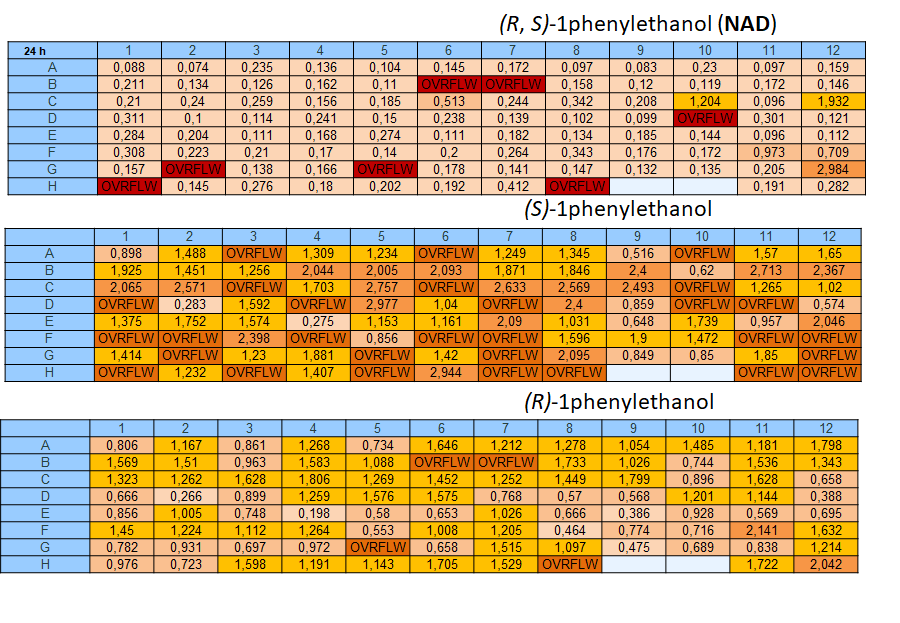


24 h

*ovrflw: absorbance values over 3 (corresponding to the limit of the instrument used for the detection

*ovrflw: absorbance values over 3 (corresponding to the limit of the instrument used for the detection

After a comparison with the plate with the addition of racemic substrate **1** and 1 mM NAD^+^ at 30 min and 24 h we can conclude that:

H1 and G2 are active towards ***S*-1**

No enzyme is active exclusively towards ***R*-1**

H8 and D10 show a stereopreference for ***S*-1** at the beginning of the reaction

G5 B6 B7 are active towards **both the enantiomers**

**Cofactor Undefined-KREDS (NADP^+^)**


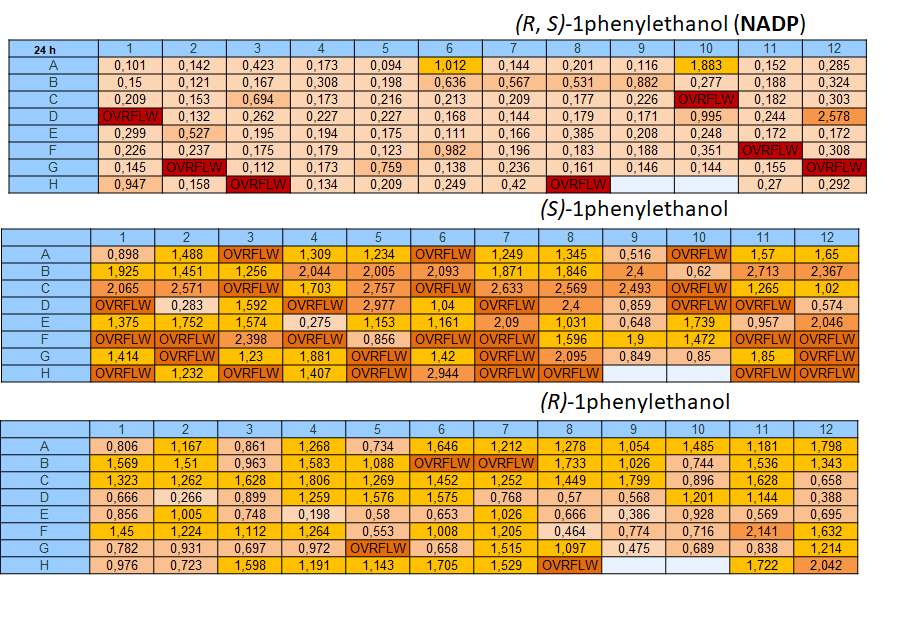


24 h


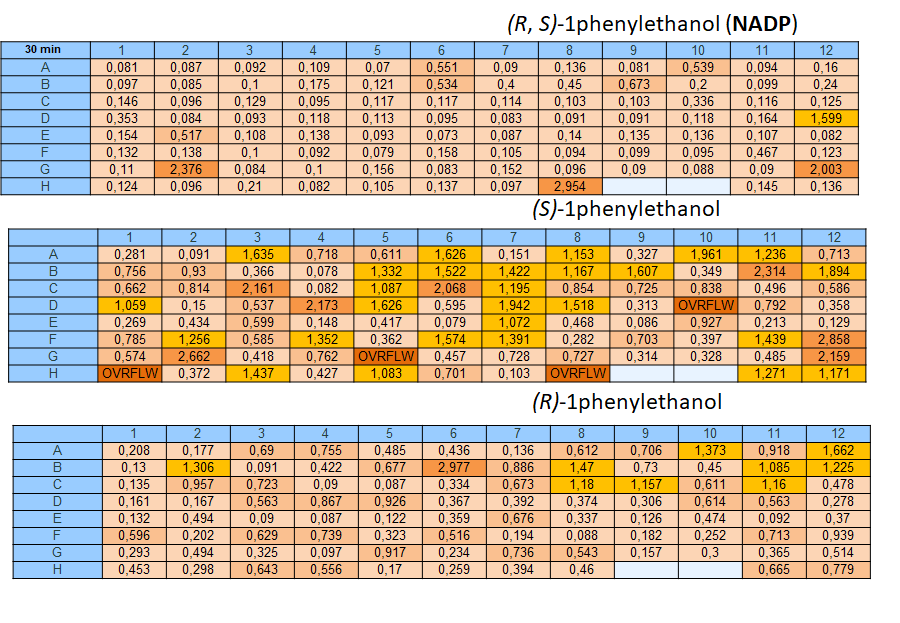


30 min


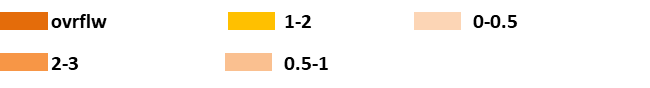


*ovrflw: absorbance values over 3 (corresponding to the limit of the instrument used for the detection

After a comparison with the plate with the addition of racemic substrate **1** and 1 mM NAD+P^+^ at 30 min and 24 h we can conclude that:

D1 is active towards ***S*-1**

No enzyme is active exclusively towards ***R*-1**

G2, H3, H8, C10, G12 show a stereopreference for *S*-A at the beginning of the reaction

F11 is active towards **both the enantiomers**

4. **HPLC and GC analysis**

The biotransformations were followed by HPLC using the described methodology in paragraph 2 or GC (for compound **C**) using the following analytical method: Agilent GC equipped with a CP-Chirasil-Dex CB column

(25m X 250 μm x 0.25 μm), with the injector temperature at 250 °C. Temperature gradient: from 80 °C to

180 °C with 5 °C/min. Retention times have been compared with commercially available products.

Substrate **1**, Rt substrate: 5.62 min; rt product: 5.99 min.

Substrate **2**, Rt substrate: 5.88 min; rt product 5.99 min.

Substrate **3**, Rt substrate: 3.39 min; rt product: 2.28 min.

Substrate **4**, Rt substrate 4.4 min; rt product 5.2 min (oxidation of the final -OH)

Substrate **5**, Rt substrate: 6.18 min; rt product: 6.87 min

Substrate **6**, Rt substrate: 4.80 min; rt product: 5.19 min

Eugenol: Rt substrate: 6.33 min, rt product: 5.01 min

Vanllyl alcohol: Rt substrate: 4.55 min; rt product: 5.16 min

**5. Alcohol Oxidases**

|  | **Enzyme** | **Abbreviation** | **Supplier** |
| --- | --- | --- | --- |
| 1 | Alcohol oxidase *Phanerochaete chrysosporium* | AOX | GECCO |
| 2 | Alditol oxidase *Acidothermus cellulotycus* | HOT-ALDO | GECCO |
| 3 | Alditol oxidase *Streptomyces coelicolor* | ALDO | GECCO |
| 4 | Chitooligosaccharide oxidase *Fusarium graminearum* | CHITO | GECCO |
| 5 | Q268R/G270E/S410R *Fusarium graminearum* | CHITO-M3 | GECCO |
| 6 | Eugenol oxidase *Rhodococcus jostii* | EUGO | GECCO |
| 7 | 5-Hydroxymethylfurfural oxidase *Methylovorus* strain | HMFO | GECCO |
| 8 | Vanillyl alcohol oxidase *Penicillium simplicissimum* | VAO | GECCO |
| 9 | 6-Hydroxy-d-nicotine oxidase *Arthrobacter nicotinovorans* | NICO | GECCO |
| 10 | Putrescine oxidase *Rhodococcus erythropolis* NCIMB 11540 | PUO | GECCO |
| 11 | Galactose oxidase *Daxtylium dendroides* | GALO | Sigma |
| 12 | Glucose oxidase *Aspergillus niger* | GLUO | Sigma |

**6. Calibration curve with increased concentration of H_2_O_2_**

Calibration curve was built by adding growing concentrations of H_2_O_2_, from 0 to 30 μM, which is the detection limit of the kit. On the left it is possible to appreciate the growing intensity of the purple color by increasing the H_2_O_2_ concentration. On the right the calibration curve based on absorbance values and corresponding H_2_O_2_ concentration


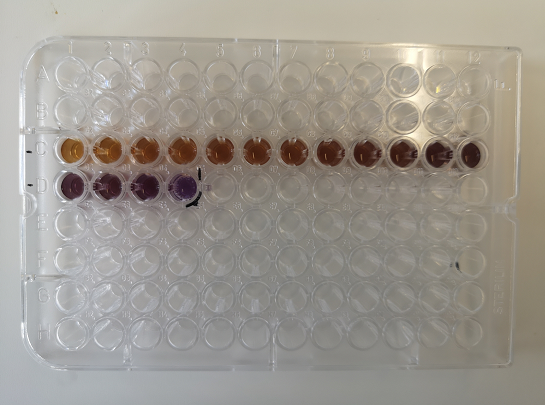


Blank

30 microM H_2_O_2_

3

10

9

12

18

24


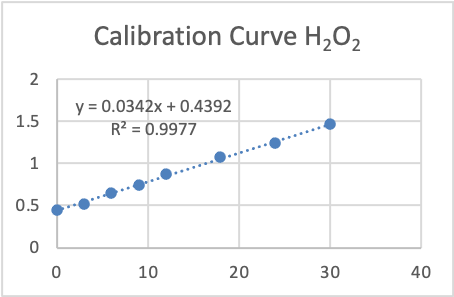


Abs

Conc (μM)

**7. LMS plate layout, table of mediators and results**

| 4 | 8 | 12 | 16 | 20 |  | 4 | 8 | 12 | 16 | 20 |  |
| --- | --- | --- | --- | --- | --- | --- | --- | --- | --- | --- | --- |
| 3 | 7 | 11 | 15 | 19 |  | 3 | 7 | 11 | 15 | 19 |  |
| 2 | 6 | 10 | 14 | 18 |  | 2 | 6 | 10 | 14 | 18 |  |
| 1 | 5 | 9 | 13 | 17 |  | 1 | 5 | 9 | 13 | 17 |  |
| 4 | 8 | 12 | 16 | 20 |  | 4 | 8 | 12 | 16 | 20 |  |
| 3 | 7 | 11 | 15 | 19 |  | 3 | 7 | 11 | 15 | 19 |  |
| 2 | 6 | 10 | 14 | 18 |  | 2 | 6 | 10 | 14 | 18 |  |
| 1 | 5 | 9 | 13 | 17 |  | 1 | 5 | 9 | 13 | 17 |  |

| pH 4.5 |  |
| --- | --- |
| pH 5.0 |  |
| pH 5.5 |  |
| pH 6.0 |  |
| B | Blank |
| 1 - 20 | Mediator |

|  | **Mediator** |
| --- | --- |
| 1 | Violuric Acid |
| 2 | TEMPO (2,2,6,6-Tetramethylpiperidin-1-yl)oxyl) |
| 3 | AZADO (2-Azaadamantane-N-oxyl) |
| 4 | Phenol Red |
| 5 | 4-hydroxy-3-methyl benzyl alcohol |
| 6 | **Water Blank** |
| 7 | Transcinnamic acid |
| 8 | *p*-Coumaric acid |
| 9 | Methyl 3-5-dimethoxy-4-hydroxybenzoate |
| 10 | Gallic Acid |
| 11 | Vanillin |
| 12 | Syringaldazine |
| 13 | Syringaldehyde |
| 14 | 2-Hydroxyanthranilic acid |
| 15 | 3,5-Dimethoxy-4-hydroxyacetophenone |
| 16 | 2,6 Dimethylphenol |
| 17 | N-hydroxyphthalimide |
| 18 | 2,2 Azino-di(3-ethylbenzthiazoline) sulfonic acid |
| 19 | Coniferyl Alcohol |
| 20 | **DMSO Blank** |

Best reaction conditions, substrate **1**:

1B (AZADO pH 4.5): 82% mc

1D (Violuric acid pH 4.5): 61% mc

1F (AZADO pH 5): 91% mc

1H (Violuric Acid pH 5): 68% mc

7B (AZADO pH 5.5): 92% mc

7D (Violuric Acid pH 5.5): 84% mc

7F (AZADO pH 6): 93% mc

7G (TEMPO pH 6): 75% mc

7H (Violuric Acid pH 6): 90% mc

11H (*N*-hydroxyphthalimide pH 6): 91% mc

Best reaction conditions, substrate **2**:

3D (Methyl 3-5-dimethoxy-4-hydroxybenzoate pH 4.5): 27% mc

7G (TEMPO pH 6): 60% mc

7C (TEMPO pH 5.5): 20% mc

9D (Methyl 3-5-dimethoxy-4-hydroxybenzoate pH 5.5): 23% mc

Best reaction conditions, substrate **3**:

B1: (AZADO pH 4.5): 65% mc

D2: (4-hydroxy-3-methyl benzyl alcohol pH 4.5): 60% mc

F2: (Transcinnamic acid pH 5): 56% mc

mc = molar conversion

No clear results have been obtained for substrate **4** and **6**, probably due over-oxidation phenomena, typically observed with the use of LMSs. No oxidation reaction was observed for substrate **5**.
